# Long-Term Cardiometabolic Outcomes in Children With Metabolically Healthy and Unhealthy Obesity

**DOI:** 10.1001/jamapediatrics.2026.0343

**Published:** 2026-03-23

**Authors:** Resthie R. Putri, Pernilla Danielsson, Emilia Hagman, Claude Marcus

**Affiliations:** 1 Department of Clinical Science, Intervention and Technology, Division of Pediatrics, Karolinska Institutet, Stockholm, Sweden; 2 Department of Medical Epidemiology and Biostatistics, Karolinska Institutet, Stockholm, Sweden

## Abstract

**Importance:**

Metabolically healthy obesity (MHO) in children has been considered a low-risk phenotype, potentially not requiring treatment. However, their long-term cardiometabolic outcomes remain unclear.

**Objective:**

To compare the occurrence of type 2 diabetes, hypertension, dyslipidemia, and mortality up to young adulthood in children with metabolically healthy obesity (MHO), metabolically unhealthy obesity (MUO), and general population peers, and to investigate the association between obesity treatment response and disease risk.

**Design, Setting, and Participants:**

This was a prospective cohort study including children undergoing obesity treatment recorded in the Swedish Childhood Obesity Treatment Register (BORIS) between 1997 and 2020 and their general population comparators, linked with national registers. Children in the cohort with obesity were aged 7 to 17 years at obesity treatment initiation and had complete cardiometabolic data. General population comparators were matched (ratio 1:5) based on sex, birth year, and residential area. Study data were analyzed from February to March 2025.

**Exposures:**

Exposures included metabolically healthy obesity (MHO), defined as the absence of high blood pressure, impaired fasting glycemia, elevated transaminases, elevated triglycerides, and low high-density lipoprotein cholesterol; otherwise, children were categorized as having metabolically unhealthy obesity (MUO).

**Main Outcomes and Measures:**

Type 2 diabetes, hypertension, dyslipidemia, and mortality up to age 30 years.

**Results:**

A total of 7275 children (median [first quartile {Q1}-third quartile {Q3}] age, 11.1 [9.1-13.5] years; 4004 male [55.0%]) were included, along with 35 636 general population comparators (median [Q1-Q3] age, 11.1 [9.1-13.5] years; 19 596 male [55.0%]). MHO at baseline was present in 3626 children (49.8%; median [Q1-Q3] age, 10.6 [8.8-12.8] years; 1981 male [54.6%]), and MUO was present in 3649 children (50.2%; median [Q1-Q3] age, 11.6 [9.4-14.0] years; 2023 male [55.4%]). By age 30 years, cumulative incidences were as follows: type 2 diabetes (MHO, 9.1%; MUO, 16.8%; general population, 0.5%), hypertension (MHO, 10.8%; MUO, 18.3%; general population, 3.7%), and dyslipidemia (MHO, 5.3%; MUO, 12.7%; general population, 0.9%). A reduction of at least 0.25 body mass index (BMI) *z* score was associated with reduced incidence rate ratio (IRR) of type 2 diabetes (IRR, 0.22; 95% CI, 0.14-0.35), hypertension (IRR, 0.56; 95% CI, 0.34-0.93), and dyslipidemia (IRR, 0.28; 95% CI, 0.14-0.57), with similar risk reduction for MHO and MUO.

**Conclusions and Relevance:**

Results of this cohort study reveal that a reduction in BMI *z* score of at least 0.25 was associated with similar risk reductions for both MHO and MUO. Children with MHO face a substantially increased cardiometabolic disease risk already as young adults compared with the general population. Hence, obesity treatment should be recommended for all children with obesity, regardless of initial metabolic status.

## Key Points

**Question:**

Do children with metabolically healthy obesity (MHO) have an increased risk in long-term cardiometabolic outcomes?

**Findings:**

A cohort of 7275 children with obesity and 35 636 general population comparators found that, by age 30 years, those with MHO had significantly higher cumulative incidences of type 2 diabetes, hypertension, and dyslipidemia compared with the general population. Weight loss was associated with lower risk of cardiometabolic outcomes.

**Meaning:**

Results suggest that children with MHO have an increased associated risk of cardiometabolic diseases up to young adulthood; pediatric obesity treatment should be offered regardless of metabolic status.

## Teaser

This cohort study evaluates if metabolically healthy obesity in children is associated with increased long-term cardiometabolic outcomes.

## Introduction

Obesity is a heterogeneous disease. For some individuals, obesity progresses rapidly with the occurrence of several cardiometabolic comorbidities, whereas others remain relatively free from such complications over time. This heterogeneity contributes to varying perspectives on its definition and management. Many organizations define obesity as a disease and advocate for early treatment.^1,2,3^ Contrarily, other experts question whether obesity should be considered as a disease or treated unless comorbidities are present.^4,5^ In addition, some argue that addressing stigma and psychosocial factors should take precedence over weight loss.^6^

A crucial aspect of this debate concerns individuals with obesity who do not exhibit overt metabolic abnormalities, sometimes classified as having *metabolically healthy obesity* (MHO), as they are suggested to experience little to no increased risk of morbidities.^7^ Consequently, weight loss in these individuals may be considered unnecessary. Although this perspective has been challenged,^8^ it has been proposed that weight-neutral support can reduce stigma for individuals with obesity.^6^ To reconcile these conflicting perspectives, a large group of experienced researchers and clinicians, in collaboration with representatives from patient organizations, formed a Lancet Commission to redefine obesity.^9^ The commission proposed a classification system that distinguishes between preclinical obesity, which is associated with minimal health risks and therefore rarely requires treatment, and clinical obesity, which is a disease defined by excess adiposity in conjunction with specific comorbidities, including but not limited to cardiometabolic comorbidities.^9^ The commission suggests a similar framework for both children and adults, with minor differences in criteria.

Already from childhood, obesity carries significantly increased disease risks across a broad spectrum, from autoimmune diseases, cancers, and cardiometabolic diseases to psychosocial and cognitive issues.^10,11,12,13^ Metabolic risk markers such as impaired fasting glycemia and elevated transaminases in childhood obesity are well known to increase the risk of developing diseases such as type 2 diabetes.^14,15^ Recent studies^16,17^ have shown that not only a substantial weight loss achieved through bariatric surgery in adolescents but also a modest weight reduction through lifestyle interventions in children and adolescents can significantly reduce the risk of developing type 2 diabetes in young adulthood.^15,18^ However, the long-term cardiometabolic risk among children with obesity who do not exhibit signs of metabolic dysfunction remains unclear.^19^ Furthermore, the potential benefits of weight reduction in children with MHO, particularly in terms of lowering the risk of future cardiometabolic disease, are yet to be established. Elucidating whether children with MHO need or benefit from obesity treatment is crucial, as the implications of the evidence would affect the treatment burden on affected children, their families, and society. Therefore, the present study aimed to

1. Compare the risk of type 2 diabetes, hypertension, dyslipidemia, and mortality in young adulthood among 3 groups: children with obesity and normal cardiometabolic risk markers (MHO), children with obesity and signs of cardiometabolic risk (termed *metabolically unhealthy obesity* [MUO]), and their peers from the general population and
2. Compare the impact of a favorable response to lifestyle-based obesity treatment on the disease risk between the groups with MHO and MUO within the pediatric obesity cohort.

### Design and Methods

#### Study Design

A cohort study of children undergoing obesity treatment enrolled in the Swedish Childhood Obesity Treatment Register (BORIS) from 1997 to 2020 and their general population comparators was conducted. In the obesity cohort, we included individuals with obesity who were aged 7 to 17 years at treatment initiation and had complete cardiometabolic data (ie, blood pressure, elevated alanine aminotransferases [ALT], fasting glucose, high-density lipoprotein [HDL] cholesterol, and triglycerides) within the first 3 months of obesity treatment. General population comparators were matched (ratio 1:5) based on sex, birth year, and residential area. Matching was performed without replacement. We excluded individuals with genetic syndromes related to obesity (eTable 1 in Supplement 1). In addition, when analyzing each outcome, outcome-specific exclusion criteria were also applied (eTable 1 in Supplement 1). Participant race and ethnicity information is not collected in Swedish national registers. As this study is based on register data, information on race or ethnicity was, therefore, not available. The study followed the Strengthening the Reporting of Observational Studies in Epidemiology (STROBE) reporting guidelines and was approved by the Regional ethical review board in Stockholm and followed the Declaration of Helsinki.

The individuals were followed up from the first visit of obesity treatment, or the corresponding matching date for the general population comparators, until the event outcome, age 30 years, death, emigration, or the end of follow-up (July 2023), whichever occurred first. The outcomes were type 2 diabetes, hypertension, dyslipidemia, and mortality.

#### Variables

Obesity was measured using body mass index (BMI) *z* score, which is standardized for age and sex, according to the International Obesity Task Force.^20^ Within the obesity cohort, obesity was categorized into class I, class II, and class III obesity, corresponding to adult BMI of 30 to 34.9 (calculated as weight in kilograms divided by height in meters squared), 35 to 39.9, and greater than or equal to 40, respectively.

The exposure, MHO in the cohort with obesity was identified in patients who did not have high blood pressure, impaired fasting glycaemia, elevated ALT level, elevated triglycerides, or low level of HDL cholesterol; otherwise, they were classified as having MUO. High blood pressure was defined as systolic and/or diastolic blood pressure greater than or equal to the 95th percentile adjusted for sex, age, and height.^21^ Impaired fasting glycemia was defined as fasting glucose greater than or equal to 110 mg/dL (to convert to millimoles per liter, multiply by 0.0555).^22^ Elevated ALT level was defined as ALT greater than or equal to 52 U/L (to convert to microkatals per liter, multiply by 0.0167) in male children and ALT greater than or equal to 44 U/L in female children.^23,24^ Elevated triglycerides were defined as triglyceride levels greater than or equal to 100 mg/dL (to convert to millimoles per liter, multiply by 0.0113) in children aged 5 to 9 years or greater than or equal to130 mg/dL in children aged 10 years or older. Low HDL cholesterol was defined as an HDL level less than 40 mg/dL (to convert to millimoles per liter, multiply by 0.0259).^25^ Additionally, we repeated the classification using lower thresholds for ALT (≥26 U/L for males and ≥22 U/L for females)^26^ and fasting glucose (≥100 mg/dL)^27^ to identify MHO in a sensitivity analysis.

The outcome, type 2 diabetes, was defined as a clinical diagnosis in specialized care or the dispensing of antidiabetic medications, according to predefined algorithms.^15^ Hypertension and dyslipidemia were similarly defined, based on clinical diagnoses in specialized care or the dispensing of antihypertensive and lipid-lowering medications, respectively. The list of diagnosis based on *International Statistical Classification of Diseases and Related Health Problems, Tenth Revision *(*ICD-10*) codes and medications based on Anatomical Therapeutic Chemical codes to identify the outcomes are reported in eTable 2 in Supplement 1.

Obesity treatment response was measured based on the BMI *z* score change between the first and last visit among individuals who underwent obesity treatment for at least 1 year. Treatment response was categorized into 3 categories: BMI *z* score reduction of 0.25 units or more, BMI *z* score increase of 0.25 units or more, and BMI *z* score change of less than 0.25 units in either direction.^18^

#### Data Sources

The primary data source was BORIS. This register records children undergoing obesity treatment across Sweden in various levels of health care, ranging from primary care to university hospitals. Clinical data during obesity treatment (such as anthropometrics, blood pressure, laboratory test results) were collected. The register was estimated to have 94% national coverage in 2023. Details about BORIS are published elsewhere.^28^

The Total Population Register (1997-2020) was used to obtain general population comparators for individuals in BORIS and to obtain data on sex and migration. Other data sources were the National Patient Register (1997-2023) to identify clinical diagnosis and medical procedures in specialized care, the National Prescribed Drug Register (2005-2023) to identify dispensing medications at all health care level, and the Cause of Death Register (1997-2023) to identify the occurrence and date of death.

### Statistical Analysis

Descriptive analyses are presented as proportion for categorical variables and median (quartile 1 [Q1]-quartile 3 [Q3]) for continuous variables. Comparison of baseline characteristics between groups was assessed using standardized mean difference (SMD). Cumulative incidence of each outcome was estimated using flexible parametric models,^29^ modeling the baseline hazard with restricted cubic splines. The number of degrees of freedom (3-4) was selected for each outcome based on model fit assessed using the bayesian information criterion. Proportional hazard assumptions were assessed based on Schoenfeld residuals and inspection of log-log survival plots.

Adjusted incidence rate of each outcome was estimated using Poisson regression with time split based on age into 5-year intervals. For each outcome, 2 models were applied. First, a model adjusted for sex and age at baseline for the whole study population. Second, a model adjusted for sex, age, and degree of obesity at baseline for the obesity cohort. The incidence rate per 10 000 person-year and its 95% CI were reported. Poisson regression was also used to compare incidence rates of each outcome between the groups with MHO and MUO and the general population comparators and to assess whether age at baseline modified these associations.

In a subgroup of individuals undergoing obesity treatment for at least 1 year, the association between BMI *z* score change and type 2 diabetes, hypertension, and dyslipidemia was assessed using Poisson regression adjusted for sex, age, and degree of obesity at baseline. Effect modification by degree of obesity and MHO in the association was assessed. Incidence rate ratio (IRR) and the 95% CI were reported. For all the regression analyses incorporating degree of obesity, class II and class III obesity were combined into a category due to limited statistical power. Data analyses were performed from February to March 2025 using Stata, version 16 (StataCorp). All *P* values were 2-sided, and *P* <.05 was considered statistically significant. Sensitivity analyses of cumulative incidence of each outcome were conducted: first, by excluding individuals who underwent bariatric surgery during follow-up and second, by applying lower ALT and fasting glucose threshold to identify MHO.

## Results

### Study Population

Among the 29 716 individuals in the cohort with obesity who were linked to Swedish national registers, 7275 (median [Q1-Q3] age, 11.1 [9.1-13.5] years; 3271 female [45.0%]; 4004 male [55.0%]) fulfilled the inclusion criteria, and 35 636 individuals (median [Q1-Q3] age, 11.1 [9.1-13.5] years; 16 040 female [45.0%]; 19 596 male [55.0%]) from the general population were selected as comparators. The median (Q1-Q3) duration of follow-up time was 8.3 (5.4-11.6) years. The median (Q1-Q3) age at the end of follow-up date was 19.5 (16.3-23.1) years. Baseline characteristics of included and excluded participants are reported in eTable 3 in Supplement 1.

Of individuals with obesity, 3626 children (49.8%; median [Q1-Q3] age, 10.6 [8.8-12.8] years; 1645 female [45.4%]; 1981 male [54.6%]) had MHO at obesity treatment initiation. MUO was present in 3649 children (50.2%; median [Q1-Q3] age, 11.6 [9.4-14.0] years; 1626 female [44.6%]; 2023 male [55.4%]). By age group at baseline, the prevalence of MHO was 59.6% in children aged 7 to 8.9 years, 51.8% in children aged 9 to 12.9 years, and 40.1% in those aged 13 years or older. Of individuals with MUO, the prevalence of elevated blood pressure at baseline was 33.6%, impaired fasting glycemia 9.0%, elevated ALT level 19.4%, low HDL level 28.7%, and elevated triglyceride level 53.4%. Characteristics of the obesity cohort are shown in Table 1.

**Table 1.** Characteristics of the Cohort With Obesity

Characteristic	Obesity cohort (N = 7275)	MUO (n = 3649)	MHO (n = 3626)	SMD^a^
Sex, No. (%)				
Female	3271 (45.0)	1626 (44.6)	1645 (45.4)	−0.02
Male	4004 (55.0)	2023 (55.4)	1981 (54.6)	
Age at baseline, median (Q1-Q3)	11.1 (9.1 to 13.5)	11.6 (9.4 to 14.0)	10.6 (8.8 to 12.8)	0.29
BMI *z *score, median (Q1-Q3)	2.75 (2.51 to 3.04)	2.82 (2.56 to 3.13)	2.68 (2.47 to 2.93)	0.36
Class I obesity, No. (%)	4603 (63.3)	2041 (55.9)	2562 (70.7)	
Class II obesity, No. (%)	1953 (26.9)	1110 (30.4)	843 (23.2)	
Class III obesity, No. (%)	719 (9.8)	498 (13.7)	221 (6.1)	
SBP, median (Q1-Q3), mm Hg	113 (105 to 120)	117 (110 to 126)	110 (104 to 116)	0.78
DBP, median (Q1-Q3), mm Hg	67 (61 to 73)	70 (63 to 75)	65 (60 to 70)	0.47
Fasting glucose, median (Q1-Q3), mg/dL	94 (88 to 99)	94 (88 to 100)	94 (88 to 97)	0.25
ALT, median (Q1-Q3), U/L	23 (19 to 32)	26 (20 to 41)	22 (17 to 28)	0.66
HDL-C, median (Q1-Q3), mg/dL	46 (39 to 54)	43 (35 to 50)	50 (43 to 58)	−0.76
Triglycerides, median (Q1-Q3), mg/dL	89 (62 to 124)	115 (80 to 159)	71 (53 to 89)	1.20

Abbreviations: ALT, alanine aminotransferase; BMI, body mass index; DBP, diastolic blood pressure; HDL-C, high-density lipoprotein cholesterol, MHO, metabolically healthy obesity; MUO, metabolically unhealthy obesity; Q, quartile; SBP, systolic blood pressure; SMD, standardized mean difference.

SI conversion factor: To convert glucose to millimoles per liter, multiply by 0.0555; ALT to microkatals per liter, multiply by 0.0167; HDL-C to millimoles per liter, multiply by 0.0259; triglycerides to millimoles per liter, multiply by 0.0113.

^a^ SMDs were calculated as (MHO − MUO).

### Incidence of Type 2 Diabetes, Hypertension, Dyslipidemia, and Mortality in MUO, MHO, and the General Population

By age 30 years, in the groups with MUO, MHO, and the general population, the cumulative incidence of type 2 diabetes was 16.8%, 9.1%, and 0.5%, respectively; the cumulative incidence of hypertension was 18.3%, 10.8%, and 3.7%, respectively; the cumulative incidence of dyslipidemia was 12.7%, 5.3%, and 0.9%, respectively; and the cumulative incidence of premature mortality was 0.8%, 0.3%, and 0.4%, respectively (Figure). In the obesity cohort, the risk for type 2 diabetes, hypertension, and dyslipidemia in the groups with MHO and MUO did not differ by age at obesity treatment initiation (*P* for interaction = .29 for type 2 diabetes, *P* for interaction = .11 for hypertension, and *P* for interaction = .22 for dyslipidemia). eFigures 1 and 2 in Supplement 1 show the risk of the investigated outcomes in individuals starting obesity treatment at age 7 to 11 years and 12 to 17 years, separately.

**Figure.**
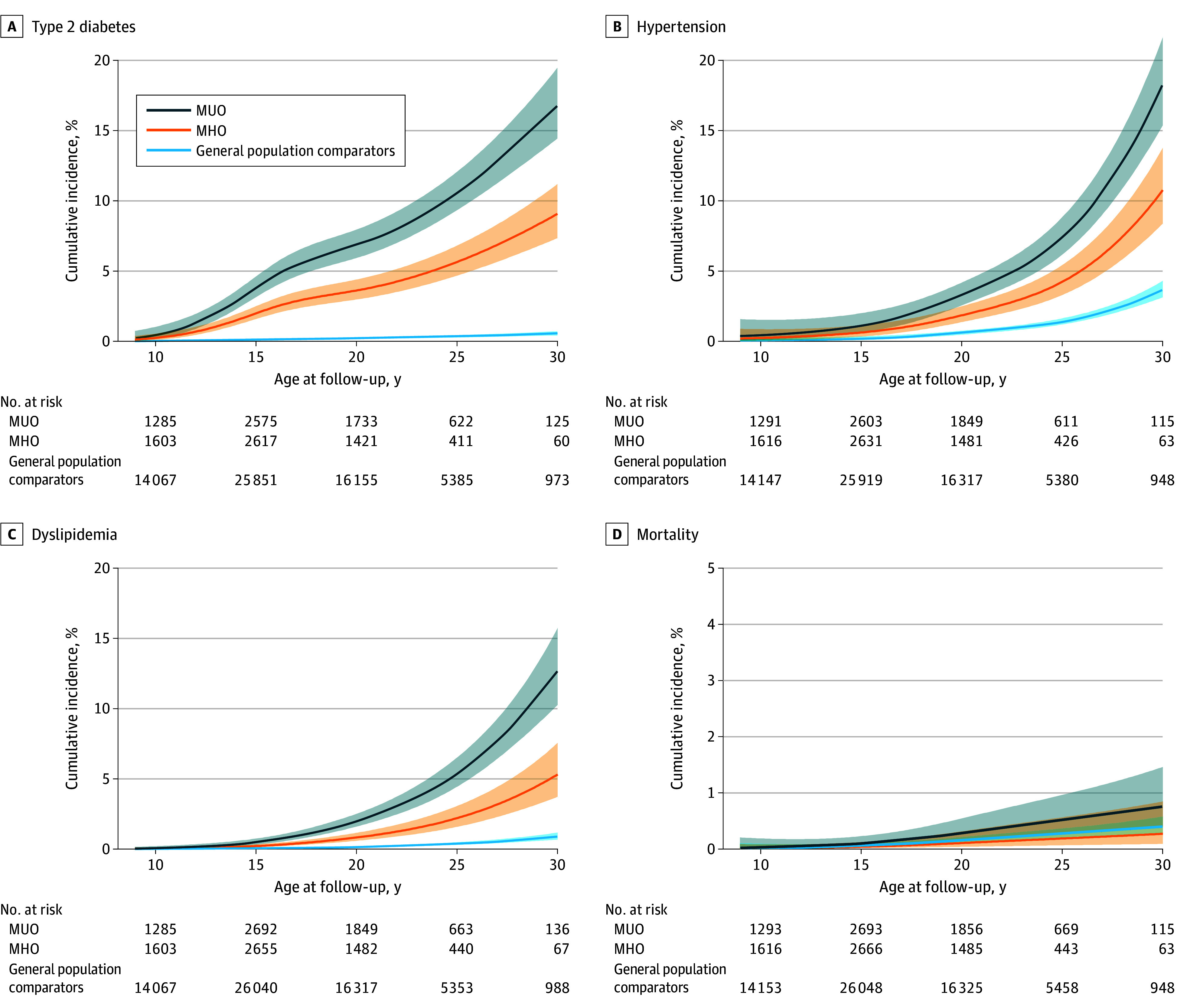
Line Graphs Showing Cumulative Incidence of Type 2 Diabetes, Hypertension, Dyslipidemia, and Premature Mortality From Age 10 to 30 Years Cumulative incidence (95% CI) of type 2 diabetes (A), hypertension (B), dyslipidemia (C), and premature mortality (D) from age 10 to 30 years in children with metabolic unhealthy obesity (MUO), metabolic healthy obesity (MHO), and general population comparators. Note the different y-scale for Figure 1D. Cumulative incidence was estimated using flexible parametric survival models.

In line with the cumulative incidence between groups, the cohort with MHO had higher adjusted incidence rate of type 2 diabetes, hypertension, and dyslipidemia compared with their general population comparators (type 2 diabetes, 36.2; 95% CI, 29.3-43.0 vs 2.1; 95% CI, 1.6-2.6; *P* < .001; hypertension, 23.2; 95% CI, 17.7-28.9 vs 8.1; 95% CI, 7.1-9.1; *P* < .001; dyslipidemia, 11.4; 95% CI, 7.6-15.2 vs 1.9; 95% CI, 1.5-2.5; *P* < .001) (Table 2). Adjusted analyses stratified by age at treatment initiation are presented in eTable 4 in Supplement 1. The incidence rate of premature mortality in MHO (1.0; 95% CI, 0.1-2.1 per 10 000 person-years) did not differ from that of the general population comparators (1.9; 95% CI, 1.5-2.5 per 10 000 person-years) (Table 2).

**Table 2.** Adjusted Incidence Rate of Type 2 Diabetes, Hypertension, Dyslipidemia, and Mortality per 10 000 Person-Years During Age 10 to 30 Years in MUO, MHO, and General Population Comparators^a^^,^^b^

Outcome	No./total No.	IR per 10 000 person-years	
		Adjusted (1)^c^	Adjusted (2)^d^
**Type 2 diabetes**			
MUO	221/3624	68.8 (59.7-77.9)	64.2 (55.6-72.8)
MHO	107/3602	36.2 (29.3-43.0)	39.1 (31.6-46.6)
General population comparators	64/35 362	2.1 (1.6-2.6)	
**Hypertension**			
MUO	147/3556	44.6 (37.3-51.9)	43.1 (36.0-50.2)
MHO	68/3586	23.2 (17.7-28.9)	24.2 (18.4-30.0)
General population comparators	254/35 469	8.1 (7.1-9.1)	
**Dyslipidemia**			
MUO	112/3644	33.7 (27.3-39.9)	32.5 (26.3-38.6)
MHO	35/3621	11.4 (7.6-15.2)	11.9 (7.9-15.9)
General population comparators	62/35 619	1.9 (1.5-2.5)	
**Mortality**			
MUO	10/3648	2.9 (1.1-4.8)	2.5 (0.9-4.0)
MHO	3/3626	1.0 (0.1-2.1)	1.1 (0.1-2.3)
General population comparators	47/35 633	1.5 (1.1-1.9)	

Abbreviations: IR, incidence rate; MHO, metabolically healthy obesity; MUO, metabolically unhealthy obesity.

^a^ Within the obesity cohort, number of individuals having at least 1 cardiometabolic disorder were 196 individuals in MHO group and 401 individuals in MUO group.

^b^ Incidence rate was estimated using Poisson regression. Stratified analyses for the outcome of premature mortality were not performed due to limited number of individuals with the outcome.

^c^ Adjusted (1): model was adjusted for sex and age at baseline.

^d^ Adjusted (2): model was adjusted for sex, age at baseline, and degree of obesity in the obesity cohort.

In the adjusted model for premature mortality (Table 3), the adjusted IRR for class II and class III obesity was 5.52 (95% CI, 1.49-20.51), whereas the adjusted IRR for MUO was 2.18 (95% CI, 0.59-8.10). A similar pattern of higher IRRs for obesity class compared with MUO was observed for the outcome of type 2 diabetes but not for hypertension or dyslipidemia (Table 3).

**Table 3.** Metabolically Unhealthy Obesity and Degree of Obesity in Relation to the Outcomes Within the Obesity Cohort

Obesity type	Type 2 diabetes		Hypertension		Dyslipidemia		Mortality	
	IRR (95% CI)	*P* value	IRR (95% CI)	*P* value	IRR (95% CI)	*P* value	IRR (95% CI)	*P* value
MUO (MHO)^a^	1.65 (1.30-2.09)	<.001	1.78 (1.32-2.38)	<.001	2.75 (1.87-4.05)	<.001	2.18 (0.59-8.10)	.24
Class II and III obesity (vs class I obesity)^b^	2.67 (2.12-3.36)	<.001	1.89 (1.43-2.49)	<.001	1.61 (1.16-2.25)	<.001	5.52 (1.49-20.51)	.01

Abbreviations: IRR, incidence rate ratio; MHO, metabolically healthy obesity; MUO, metabolically unhealthy obesity.

^a^ IRR was estimated using Poisson regression adjusted for sex, age, and degree of obesity at baseline.

^b^ IRR was estimated using Poisson regression adjusted for sex, age, and MUO at baseline.

### Association Between Response to Pediatric Obesity Treatment and the Risk of Investigated Outcomes

The association between pediatric obesity treatment and type 2 diabetes, hypertension, and dyslipidemia was assessed in a subgroup of individuals in the obesity cohort who underwent treatment for at least 1 year (n = 2532). Reduction in BMI *z *score of at least 0.25 units was associated with lower risk of type 2 diabetes (adjusted IRR, 0.22; 95% CI, 0.14-0.35), hypertension (adjusted IRR, 0.56; 95% CI, 0.34-0.93), and dyslipidemia (adjusted IRR, 0.28; 95% CI, 0.14-0.57), compared with the group with increased BMI *z *score as the reference. The association between treatment response and the outcomes did not differ between MHO and MUO (*P* for interaction = .13), or between obesity class (*P* for interaction = .59).

Additionally, a BMI *z *score change of less than 0.25 units in either direction was also associated with lower risk of type 2 diabetes (adjusted IRR, 0.51; 95% CI, 0.37-0.70), hypertension (adjusted IRR, 0.57; 95% CI, 0.36-0.90), and dyslipidemia (adjusted IRR, 0.51; 95% CI, 0.30-0.86), compared with the group with an increase of 0.25 BMI *z* score units or more.

### Sensitivity Analyses

After excluding individuals who underwent bariatric surgery during follow-up time (n = 183), the cumulative incidence of type 2 diabetes, hypertension, dyslipidemia, and premature mortality in MUO, MHO, and general population comparators was similar as the main results (eFigure 3 in Supplement 1). Likewise, when lower thresholds of ALT and fasting glucose were applied to define MHO, the patterns of cumulative incidence remained consistent with the main findings (eFigure 4 in Supplement 1).

## Discussion

This cohort study confirms the association between obesity in children aged 7 to 17 years and the development of type 2 diabetes, hypertension, and dyslipidemia disease before 30 years of age. Although children with MHO had a lower risk than those with MUO to develop the outcomes, they still faced a substantial higher risk than their peers in the general population. Additionally, obesity itself has a major impact, as even the group with obesity class I and no metabolic disturbances at baseline had a markedly enhanced risk of the outcomes in young adulthood compared with the general population comparators. However, during adolescence, the absolute risk of the outcomes remains modest, consistent with evidence that obesity-related comorbidities may take decades to manifest.^11^ Furthermore, although we have previously shown that weight loss over 1 to 2 years of obesity treatment reduces the long-term risk of cardiometabolic diseases,^18^ the present study found that groups with MUO and MHO had similar a long-term protective effect of weight loss during childhood on their future risk of type 2 diabetes, hypertension, and dyslipidemia.

In adults, MHO is defined as obesity in the absence of metabolic aberrations^30^ and has been suggested to have a minimal risk of obesity-related conditions.^31^ However, MHO has been demonstrated as a transient state, and within 10 years, the risk of cardiometabolic diseases is higher for MHO compared with normal-weight individuals.^32^ Despite that, the Lancet Commission concluded that individuals with obesity but without apparent organ dysfunction had sufficiently low medical risks that obesity treatment was generally not warranted.^9^

There is a marked variation in the long-term development of obesity-related morbidities among individuals with obesity. A considerable proportion of adults with obesity do not develop obesity-related diseases within 10 to 20 years of follow-up.^33^ A similar pattern has been observed in children.^34^ However, it remains challenging to predict which individuals are at low risk and may derive limited health benefits from weight reduction. Although currently used metabolic risk markers can offer some indications, they are not sufficient to discriminate between healthy and unhealthy obesity among children and adolescents with obesity. Exploring predictive markers for progression from MHO to MUO would be an important research area. Using precision medicine tools, we will most likely be able to identify children with truly low-risk obesity in the future.

Age appears to play a crucial role in the association between MHO and the progression of obesity-related diseases. Young adults with MHO have an increased risk of cardiovascular diseases compared with the general population, although this risk is considerably lower than for those with MUO.^14,15^ Conversely, middle-aged adults with MHO have been observed to have a slightly better life expectancy in later life than adults with normal weight.^35^ The present study indicates that even in the absence of early metabolic disturbances, children with obesity still face a markedly elevated long-term risk for cardiometabolic diseases compared with the general population. Although the future risk of cardiometabolic diseases varies among those with and without affected metabolic markers, the most substantial difference is observed when comparing the pediatric obesity cohort with their peers in the general population.

### Strengths and Limitations

We have used real-world individual-level Swedish nationwide data with follow-up spanning from childhood to adulthood. This strengthens the internal and external validity of the findings. Nevertheless, this study has some limitations. The outcomes investigated (apart from mortality) are clinically silent conditions, and the present study is restricted to cases that have been diagnosed or treated. Moreover, data on the occurrence and timing of transitions from MHO to MUO before outcomes were lacking. Additionally, only a small portion of the children in the cohort reached the age of 30 years, which limits the ability to capture later-onset of the outcome events. The small number of mortality events reduce precision and should be interpreted carefully. Furthermore, as height and weight data from the general population were lacking, this group should not be interpreted as a normal weight group, but rather as reflecting the full BMI distribution of the general population. Therefore, the effects of obesity on future health may be underestimated. In addition, data on weight development in adulthood was not available.

## Conclusions

In conclusion, in this cohort study, results suggest that children aged 7 to 17 years with metabolically healthy obesity at treatment initiation have an increased associated long-term risk of type 2 diabetes, hypertension, and dyslipidemia compared with their peers in the general population. Reduction in BMI *z *score in pediatric obesity treatment was associated with reduced cardiometabolic risk in children with metabolically healthy and unhealthy obesity to the same extent. Therefore, treatment should also be recommended for children with obesity who appear metabolically healthy.

## Notes

Supplement 1. **eTable 1.** Exclusion Criteria **eTable 2.** Codes for Identifying Disease Outcomes and Surgical Procedure **eTable 3.** Comparison of Characteristics Individuals in Obesity Cohort Included vs Excluded From the Study **eFigure 1.** Cumulative Incidence of Cardiometabolic Disorders in Individuals Starting Obesity Treatment at Age 7-11 Years and Their Matched General Population Comparators **eFigure 2.** Cumulative Incidence of Cardiometabolic Disorders in Individuals Starting Obesity Treatment at Age 12-17 Years and Their Matched General Population Comparators **eTable 4.** Adjusted Incidence Rate of Type 2 Diabetes, Hypertension, and Dyslipidemia per 10 000 Person-Years During Age 10-30 Years in MUO, MHO, and General Population Comparators **eFigure 3.** Cumulative Incidence of Cardiometabolic Disorders After Excluding Individuals Undergoing Bariatric Surgery **eFigure 4.** Cumulative Incidence of Cardiometabolic Disorders, With MHO Defined Using Lower ALT and Fasting Glucose Thresholds

Supplement 2.
Data Sharing Statement.

## References

[1] Hampl SE, Hassink SG, Skinner AC, . Clinical practice guideline for the evaluation and treatment of children and adolescents with obesity. Pediatrics. 2023;151(2):e2022060640. doi:10.1542/peds.2022-060640 36622115

[2] Gaskin CJ, Cooper K, Stephens LD, Peeters A, Salmon J, Porter J. Clinical practice guidelines for the management of overweight and obesity published internationally: a scoping review. Obes Rev. 2024;25(5):e13700. doi:10.1111/obr.13700 38296655

[3] Busetto L, Dicker D, Frühbeck G, . A new framework for the diagnosis, staging and management of obesity in adults. Nat Med. 2024;30(9):2395-2399. doi:10.1038/s41591-024-03095-3 38969880

[4] Luli M, Yeo G, Farrell E, . The implications of defining obesity as a disease: a report from the Association for the Study of Obesity 2021 annual conference. EClinicalMedicine. 2023;58:101962. doi:10.1016/j.eclinm.2023.101962 37090435 PMC10119881

[5] Steele M, Finucane FM. Philosophically, is obesity really a disease? Obes Rev. 2023;24(8):e13590. doi:10.1111/obr.13590 37279872

[6] Heitmann BL, Køster-Rasmussen R, Meyer LB, . Debating weight loss vs weight neutral strategies for improvements of health. Curr Obes Rep. 2024;13(4):832-842. doi:10.1007/s13679-024-00587-8 39289256 PMC11522117

[7] Schulze MB, Stefan N. Metabolically healthy obesity: from epidemiology and mechanisms to clinical implications. Nat Rev Endocrinol. 2024;20(11):633-646. doi:10.1038/s41574-024-01008-5 38937638

[8] Tanriover C, Copur S, Gaipov A, . Metabolically healthy obesity: misleading phrase or healthy phenotype? Eur J Intern Med. 2023;111:5-20. doi:10.1016/j.ejim.2023.02.025 36890010

[9] Rubino F, Cummings DE, Eckel RH, . Definition and diagnostic criteria of clinical obesity. Lancet Diabetes Endocrinol. 2025;13(3):221-262. doi:10.1016/S2213-8587(24)00316-4 39824205 PMC11870235

[10] Lister NB, Baur LA, Felix JF, . Child and adolescent obesity. Nat Rev Dis Primers. 2023;9(1):24. doi:10.1038/s41572-023-00435-4 37202378

[11] Marcus C, Danielsson P, Hagman E. Pediatric obesity—long-term consequences and effect of weight loss. J Intern Med. 2022;292(6):870-891. doi:10.1111/joim.13547 35883220 PMC9805112

[12] Hagman E, Putri RR, Danielsson P, Marcus C. Pediatric obesity and the risk of multiple sclerosis: a nationwide prospective cohort study. Int J Obes (Lond). 2025;49(6):1031-1036. doi:10.1038/s41366-025-01727-3 39885338 PMC12158760

[13] Wiedemann UCH, van den Akker ELT, Barber TM, . Early-onset of obesity model: impact of early-onset obesity on comorbidity risk and life expectancy. Obes Facts. Published online November 14, 2025. doi:10.1159/000549499 41237076 PMC12695130

[14] Hagman E, Danielsson P, Brandt L, Ekbom A, Marcus C. Association between impaired fasting glycemia in pediatric obesity and type 2 diabetes in young adulthood. Nutr Diabetes. 2016;6(8):e227. doi:10.1038/nutd.2016.34 27548712 PMC5022148

[15] Putri RR, Casswall T, Danielsson P, Marcus C, Hagman E. Steatotic liver disease in pediatric obesity and increased risk for youth-onset type 2 diabetes. Diabetes Care. 2024;47(12):2196-2204. doi:10.2337/dc24-1236 39373987 PMC11655408

[16] Ryder JR, Jenkins TM, Xie C, . Ten-year outcomes after bariatric surgery in adolescents. N Engl J Med. 2024;391(17):1656-1658. doi:10.1056/NEJMc2404054 39476348 PMC11753263

[17] Inge TH, Courcoulas AP, Jenkins TM, ; Teen–LABS Consortium. Five-year outcomes of gastric bypass in adolescents as compared with adults. N Engl J Med. 2019;380(22):2136-2145. doi:10.1056/NEJMoa1813909 31116917 PMC7345847

[18] Putri RR, Danielsson P, Ekström N, . Effect of pediatric obesity treatment on long-term health. JAMA Pediatr. 2025;179(3):302-309. doi:10.1001/jamapediatrics.2024.5552 39836390 PMC11877215

[19] Vukovic R, Dos Santos TJ, Ybarra M, Atar M. Children with metabolically healthy obesity: a review. Front Endocrinol (Lausanne). 2019;10:865. doi:10.3389/fendo.2019.00865 31920976 PMC6914809

[20] Cole TJ, Lobstein T. Extended international (IOTF) body mass index cut-offs for thinness, overweight and obesity. Pediatr Obes. 2012;7(4):284-294. doi:10.1111/j.2047-6310.2012.00064.x 22715120

[21] National High Blood Pressure Education Program Working Group on High Blood Pressure in Children and Adolescents. The fourth report on the diagnosis, evaluation, and treatment of high blood pressure in children and adolescents. Pediatrics. 2004;114(2 suppl 4th report):555-576. 15286277

[22] World Health Organization. Definition and diagnosis of diabetes mellitus and intermediate hyperglycemia: report of a WHO/IDF consultation. Accessed November 28, 2013. https://iris.who.int/items/e6e9ee5a-cacf-4278-97be-c6a967f6520a

[23] Vos MB, Abrams SH, Barlow SE, . NASPGHAN clinical practice guideline for the diagnosis and treatment of nonalcoholic fatty liver disease in children: recommendations from the expert committee on NAFLD (ECON) and the North American Society of Pediatric Gastroenterology, Hepatology, and Nutrition (NASPGHAN). J Pediatr Gastroenterol Nutr. 2017;64(2):319-334. doi:10.1097/MPG.0000000000001482 28107283 PMC5413933

[24] Swedish Association for Pediatric Gastroenterology, Hepatology, and Nutrition. Investigation and processing of fatty liver disease in children and adolescents with overweight and obesity. Article in Swedish. Accessed December 16, 2025. https://gastro.barnlakarforeningen.se/wp-content/uploads/sites/10/2021/10/utredning-och-handlaggning-av-fettleversjukdom-hos-barn-och-ungdomar-med-overvikt-och-fetma.pdf

[25] Expert Panel on Integrated Guidelines for Cardiovascular Health and Risk Reduction in Children and Adolescents; National Heart, Lung, and Blood Institute. Expert panel on integrated guidelines for cardiovascular health and risk reduction in children and adolescents: summary report. Pediatrics. 2011;128 suppl 5(suppl 5):S213-S256. doi:10.1542/peds.2009-2107C 22084329 PMC4536582

[26] Schwimmer JB, Dunn W, Norman GJ, . SAFETY study: alanine aminotransferase cutoff values are set too high for reliable detection of pediatric chronic liver disease. Gastroenterology. 2010;138(4):1357-1364, 1364.e1-2. doi:10.1053/j.gastro.2009.12.052 20064512 PMC2846968

[27] American Diabetes Association Professional Practice Committee. 2. Diagnosis and classification of diabetes: standards of care in diabetes-2025. Diabetes Care. 2025;48(1)(suppl 1):S27-S49. doi:10.2337/dc25-S002 39651986 PMC11635041

[28] Hagman E, Danielsson P, Lindberg L, Marcus C; BORIS Steering Committee. Pediatric obesity treatment during 14 years in Sweden: lessons from the swedish childhood obesity treatment register-BORIS. Pediatr Obes. 2020;15(7):e12626. doi:10.1111/ijpo.12626 32074662

[29] Lambert PC, Royston P. Further development of flexible parametric models for survival analysis. Stata J. 2009;9(2):265-290. doi:10.1177/1536867X0900900206

[30] Magkos F. Metabolically healthy obesity: what’s in a name? Am J Clin Nutr. 2019;110(3):533-539. doi:10.1093/ajcn/nqz133 31240297

[31] Stefan N, Häring HU, Hu FB, Schulze MB. Metabolically healthy obesity: epidemiology, mechanisms, and clinical implications. Lancet Diabetes Endocrinol. 2013;1(2):152-162. doi:10.1016/S2213-8587(13)70062-7 24622321

[32] Lin H, Zhang L, Zheng R, Zheng Y. The prevalence, metabolic risk and effects of lifestyle intervention for metabolically healthy obesity: a systematic review and meta-analysis: a PRISMA-compliant article. Medicine (Baltimore). 2017;96(47):e8838. doi:10.1097/MD.0000000000008838 29381992 PMC5708991

[33] Mørkedal B, Vatten LJ, Romundstad PR, Laugsand LE, Janszky I. Risk of myocardial infarction and heart failure among metabolically healthy but obese individuals: HUNT (Nord-Trøndelag Health Study), Norway. J Am Coll Cardiol. 2014;63(11):1071-1078. doi:10.1016/j.jacc.2013.11.035 24345592

[34] Baker JL, Olsen LW, Sørensen TI. Childhood body mass index and the risk of coronary heart disease in adulthood. N Engl J Med. 2007;357(23):2329-2337. doi:10.1056/NEJMoa072515 18057335 PMC3062903

[35] Ler P, Li X, Hassing LB, . Independent and joint effects of body mass index and metabolic health in mid- and late-life on all-cause mortality: a cohort study from the Swedish Twin Registry with a mean follow-up of 13 Years. BMC Public Health. 2022;22(1):718. doi:10.1186/s12889-022-13082-3 35410261 PMC9004188

