# Supplementary material for: Long-Term Cardiometabolic Outcomes in Children With Metabolically Healthy and Unhealthy Obesity
Source: JAMA Pediatr. 2026 Mar 23;180(7):750–7. doi: 10.1001/jamapediatrics.2026.0343 (PMC13010205; doi:10.1001/jamapediatrics.2026.0343)
Supplement: Supplement 1. — eTable 1. Exclusion Criteria eTable 2. Codes for Identifying Disease Outcomes and Surgical Procedure eTable 3. Comparison of Characteristics Individuals in Obesity Cohort Included vs Excluded From the Study eFigure 1. Cumulative Incidence of Cardiometabolic Disorders in Individuals Starting Obesity Treatment at Age 7-11 Years and Their Matched General Population Comparators eFigure 2. Cumulative Incidence of Cardiometabolic Disorders in Individuals Starting Obesity Treatment at Age 12-17 Years and Their Matched General Population Comparators eTable 4. Adjusted Incidence Rate of Type 2 Diabetes, Hypertension, and Dyslipidemia per 10 000 Person-Years During Age 10-30 Years in MUO, MHO, and General Population Comparators eFigure 3. Cumulative Incidence of Cardiometabolic Disorders After Excluding Individuals Undergoing Bariatric Surgery eFigure 4. Cumulative Incidence of Cardiometabolic Disorders, With MHO Defined Using Lower ALT and Fasting Glucose Thresholds [file jamapediatr-e260343-s001.pdf]

Supplementary Online Content

Putri RR, Danielsson P, Hagman E, Marcus C. Long-term cardiometabolic outcomes in children with metabolically healthy and unhealthy obesity. *JAMA Pediatr*. Published online March 23, 2026. doi:10.1001/jamapediatrics.2026.0343

eTable 1. Exclusion Criteria

eTable 2. Codes for Identifying Disease Outcomes and Surgical Procedure

eTable 3. Comparison of Characteristics Individuals in Obesity Cohort Included vs Excluded From the Study

eFigure 1. Cumulative Incidence of Cardiometabolic Disorders in Individuals Starting Obesity Treatment at Age 7-11 Years and Their Matched General Population Comparators

eFigure 2. Cumulative Incidence of Cardiometabolic Disorders in Individuals Starting Obesity Treatment at Age 12-17 Years and Their Matched General Population Comparators

eTable 4. Adjusted Incidence Rate of Type 2 Diabetes, Hypertension, and Dyslipidemia per 10 000 Person-Years During Age 10-30 Years in MUO, MHO, and General Population Comparators

eFigure 3. Cumulative Incidence of Cardiometabolic Disorders After Excluding Individuals Undergoing Bariatric Surgery

eFigure 4. Cumulative Incidence of Cardiometabolic Disorders, With MHO Defined Using Lower ALT and Fasting Glucose Thresholds

This supplementary material has been provided by the authors to give readers additional information about their work.

© 2026 Putri RR et al. *JAMA Pediatrics*.

eTable 1. Exclusion criteria

**General exclusion criteria**	**ICD-10 codes**
Alagille syndrome	Q44.7
Down syndrome	Q90
Fragile X syndrome	Q99.2
Klinefelter syndrome	Q98
Laurence-Moon-Biedl syndrome	Q87.8B
Noonan syndrome	Q87.1E
Prader-Willi syndrome	Q87.1F
Silver-Russel syndrome	Q87.1G
Turner syndrome	Q96
**Outcome-specific exclusion criteria**	**ICD-10 codes**
Outcome: type 2 diabetes	
Type 1 diabetes	E10
Pre-existing type 1 diabetes mellitus, in pregnancy, childbirth and the puerperium	O24.0
Maturity onset of diabetes in the young (MODY)	E13
Outcome: hypertension	
Secondary hypertension	I15
Hyperaldosteronism	E26
Benign neoplasm of adrenal gland	D35
Malignant neoplasm of medulla of adrenal gland	C74.1
Outcome: dyslipidemia	
Disorders of branched-chain amino-acid metabolism and fatty-acid metabolism	E71
Disorders of sphingolipid metabolism and other lipid storage disorders	E75
Hyperchylomicronemia	E78.3

© 2026 Putri RR et al. *JAMA Pediatrics*.

eTable 2. Codes for identifying disease outcomes and surgical procedure

Description	International Classification of Diseases, tenth revision (ICD-10) codes	Anatomical Therapeutic Chemical (ATC) codes
Outcome: type 2 diabetes		
Type 2 diabetes	E11	
Biguanides		A10BA
Sulfonylureas		A10BB
Sulfonamides (heterocyclic)		A10BC
Combinations of oral blood glucose lowering drugs		A10BD
Alpha glucosidase inhibitors		A10BF
Thiazolidinediones		A10BG
Dipeptidyl peptidase 4 (DPP-4) inhibitors		A10BH
Glucagon-like peptide-1 receptor (GLP-1) analogues		A10BJ
Sodium-glucose co-transporter 2 (SGLT2) inhibitors		A10BK
Other blood glucose lowering drugs, excluding insulins		A10BX
Insulin		A10A
Outcome: hypertension		
Essential hypertension	I10	
Hypertensive heart disease	I11	
Hypertensive chronic kidney disease	I12	
Hypertensive heart and chronic kidney disease	I13	
Pre-existing essential hypertension complicating pregnancy	O10	
Agents acting on arteriolar smooth muscle		C02D
Antihypertensives and diuretics in combination		C02L
Diuretics		C03
Calcium channel blockers		C08
Agents acting on the renin-angiotensin system		C09
Outcome: dyslipidemia		
Pure hypercholesterolemia	E78.0	
Pure hyperglyceridemia	E78.1	
Mixed hyperlipidemia	E78.2	
Hyperlipidemia, unspecified	E78.5	
β-Hydroxy β-methylglutaryl-CoA reductase inhibitors		C10AA
Fibrates		C10AB
Combinations of various lipid modifying agents		C10BA
Nicotinic acid and derivatives		C10AD
Other lipid modifying agents including ezetimibe		C10AX
Weight loss bariatric surgery (for sensitivity analysis): ICD-10 code E66 (obesity) and procedure code JDF (gastric restrictive surgery)		

© 2026 Putri RR et al. *JAMA Pediatrics*.

eTable 3. Comparison of characteristics individuals in obesity cohort included vs excluded from the study.

	Included n = 7 275	Excluded n = 22 441	SMD
Boys, n (%)	4 004 (55.0)	11981 (51.9)	0.06
Age at baseline, median (Q1, Q3)	11.1 (9.1, 13.5)	9.9 (6.9, 12.9)	-0.40
BMI Z-score, median (Q1, Q3)	2.75 (2.51, 3.04)	2.76 (2.43, 3.14)	0.04
Level of care, n (%)			0.31
University hospital	1 098 (15.1)	5 241 (22.7)	
Pediatric specialized care*	6 174 (84.9)	16 633 (74.9)	
Primary care	<10 (<1%)	567 (2.4)	

Abbreviations: Q1, first quartile; Q3, third quartile; SMD, standardized mean difference
* Including pediatric clinics (barnklinik in Swedish) and pediatric ward clinics (barnmottagning in Swedish)

© 2026 Putri RR et al. *JAMA Pediatrics*.

eFigure 1 Cumulative incidence of cardiometabolic disorders in individuals starting obesity treatment at age 7-11 years and their matched general population comparators.
Dashed, solid, and dotted lines represent MUO, MHO, and general population comparators, respectively. Mortality was not estimated due to limited numbers of events.

A. Type 2 diabetes as the outcome

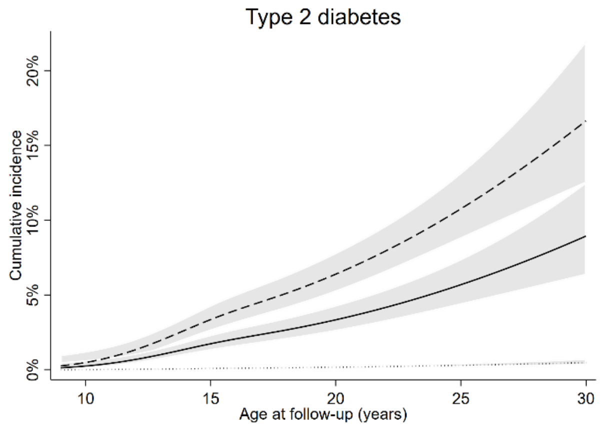

B. Hypertension as the outcome

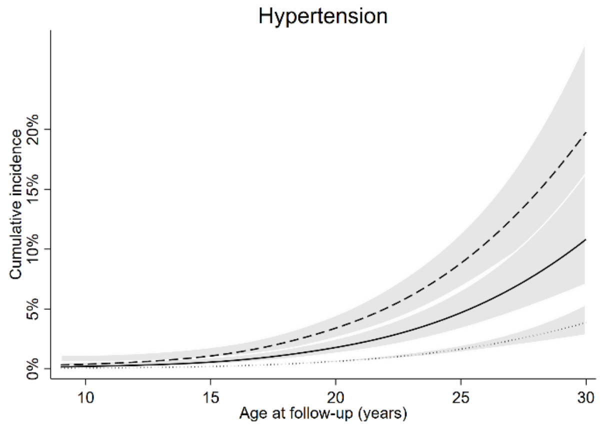

C. Dyslipidemia as the outcome

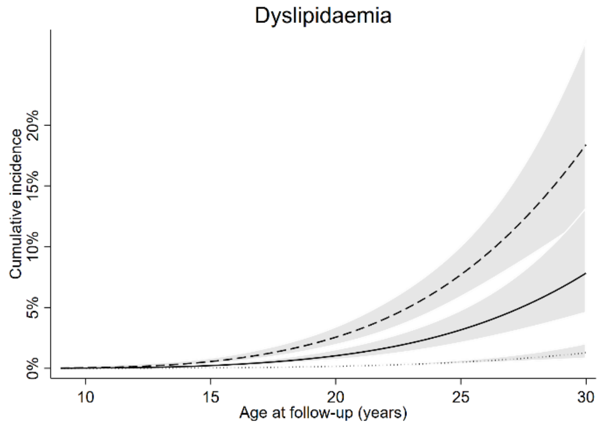

© 2026 Putri RR et al. *JAMA Pediatrics*.

e**Figure 2.** Cumulative incidence of cardiometabolic disorders in individuals starting obesity treatment at age 12-17 years and their matched general population comparators.
Dashed, solid, and dotted lines represent MUO, MHO, and general population comparators, respectively. Mortality was not estimated due to limited numbers of events.

A. Type 2 diabetes as the outcome

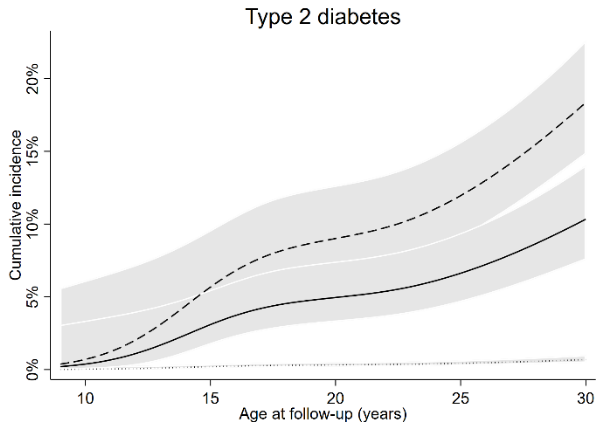

B. Hypertension as the outcome

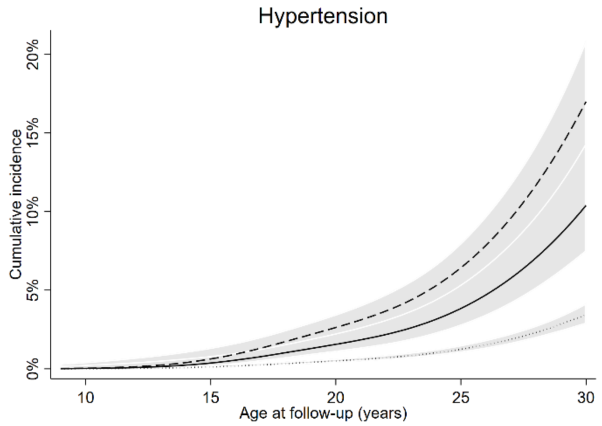

C. Dyslipidemia as the outcome

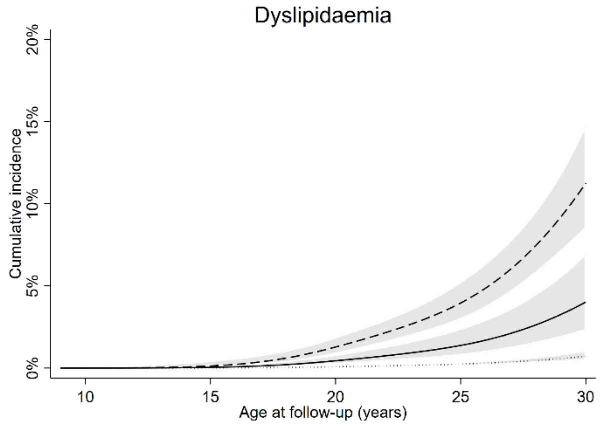

© 2026 Putri RR et al. *JAMA Pediatrics*.

eTable 4. Adjusted incidence rate of type 2 diabetes, hypertension, and dyslipidemia per 10 000 person-years during age 10-30 years in MUO, MHO, and general population comparators.

	Age 7-11 years at baseline			Age 12-17 years at baseline		
	n/ N	IR per 10000 person-years		n/ N	IR per 10000 person-years	
		Adjusted (1)	Adjusted (2)		Adjusted (1)	Adjusted (2)
Type 2 diabetes						
MUO	102/ 1973	57.5 (46.3 - 68.7)	52.4 (42.1 - 62.7)	119/ 1651	86.1 (70.5 - 101.7)	80.8 (66.1 - 95.5)
MHO	61/ 2415	30.2 (22.6 - 27.8)	32.8 (24.5 - 41.0)	46/ 1187	44.5 (31.6 - 57.4)	47.7 (33.8 - 61.6)
General population comparators	29/ 21452	1.5 (1.0 - 2.1)		35/ 13910	2.8 (1.9 - 3.8)	
Hypertension						
MUO	58/ 1930	32.9 (24.4 - 41.5)	32.4 (23.9 - 40.9)	89/1626	63.5 (50.3 - 76.7)	60.3 (47.6 - 73.0)
MHO	33/ 2368	16.0 (10.6 - 21.5)	16.4 (10.8 - 22.0)	35/ 1150	33.2 (22.2 - 44.3)	34.7 (23.1 - 46.2)
General population comparators	110/ 21485	5.8 (4.7 - 6.9)		144/ 13840	11.6 (9.7 - 13.4)	
Dyslipidemia						
MUO	52/1975	28.8 (20.9 - 36.7)	27.8 (20.1 - 35.5)	60/ 1669	30.0 (29.8 - 50.1)	38.1 (28.3 - 47.8)
MHO	22/ 2428	10.6 (6.2 - 15.0)	10.9 (6.3 - 15.5)	13/ 1193	12.7 (5.8 - 19.6)	13.9 (6.3 - 21.6)
General population comparators	33/ 21594	1.7 (1.1 - 2.3)		29/ 14025	2.3 (1.5 - 3.2)	

Abbreviations: CI, confidence interval; MHO, metabolically healthy obesity; MUO, metabolically unhealthy obesity.

Adjusted (1): the model was adjusted for sex and age at baseline. Adjusted (2): the model was adjusted for sex, age at baseline, and degree of obesity in the obesity cohort.

Incidence rate was estimated using Poisson regression. Stratified analyses for the outcome of premature mortality were not performed due to limited number of individuals with the outcome.

© 2026 Putri RR et al. *JAMA Pediatrics*.

eFigure 3. Cumulative incidence of cardiometabolic disorders after excluding individuals undergoing bariatric surgery.

Dashed, solid, and dotted lines represent MUO, MHO, and general population comparators, respectively.

A. Type 2 diabetes as the outcome

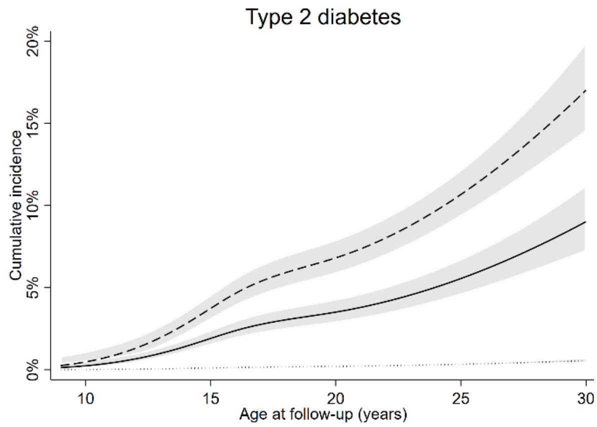

B. Hypertension as the outcome

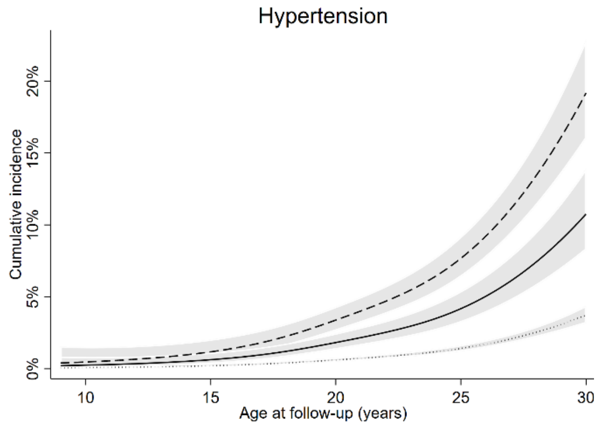

C. Dyslipidemia as the outcome

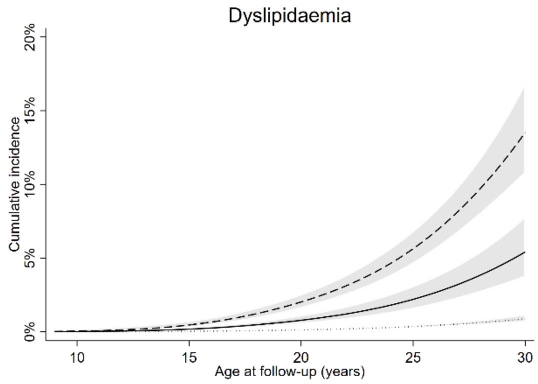

D. Mortality as the outcome

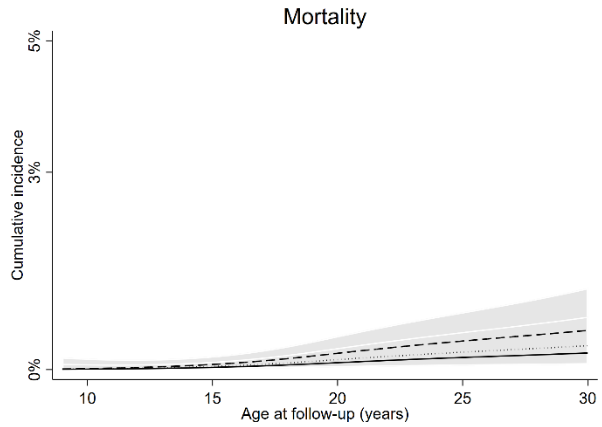

© 2026 Putri RR et al. *JAMA Pediatrics*.

eFigure 4. Cumulative incidence of cardiometabolic disorders, with MHO defined using lower ALT and fasting glucose thresholds.

Using lower ALT threshold (26 U/L for boys and 22 U/L for girls) and lower fasting glycemia threshold (5.6 mmol/L), 28.9% (n = 2103) of the obesity cohort were classified into MHO group.

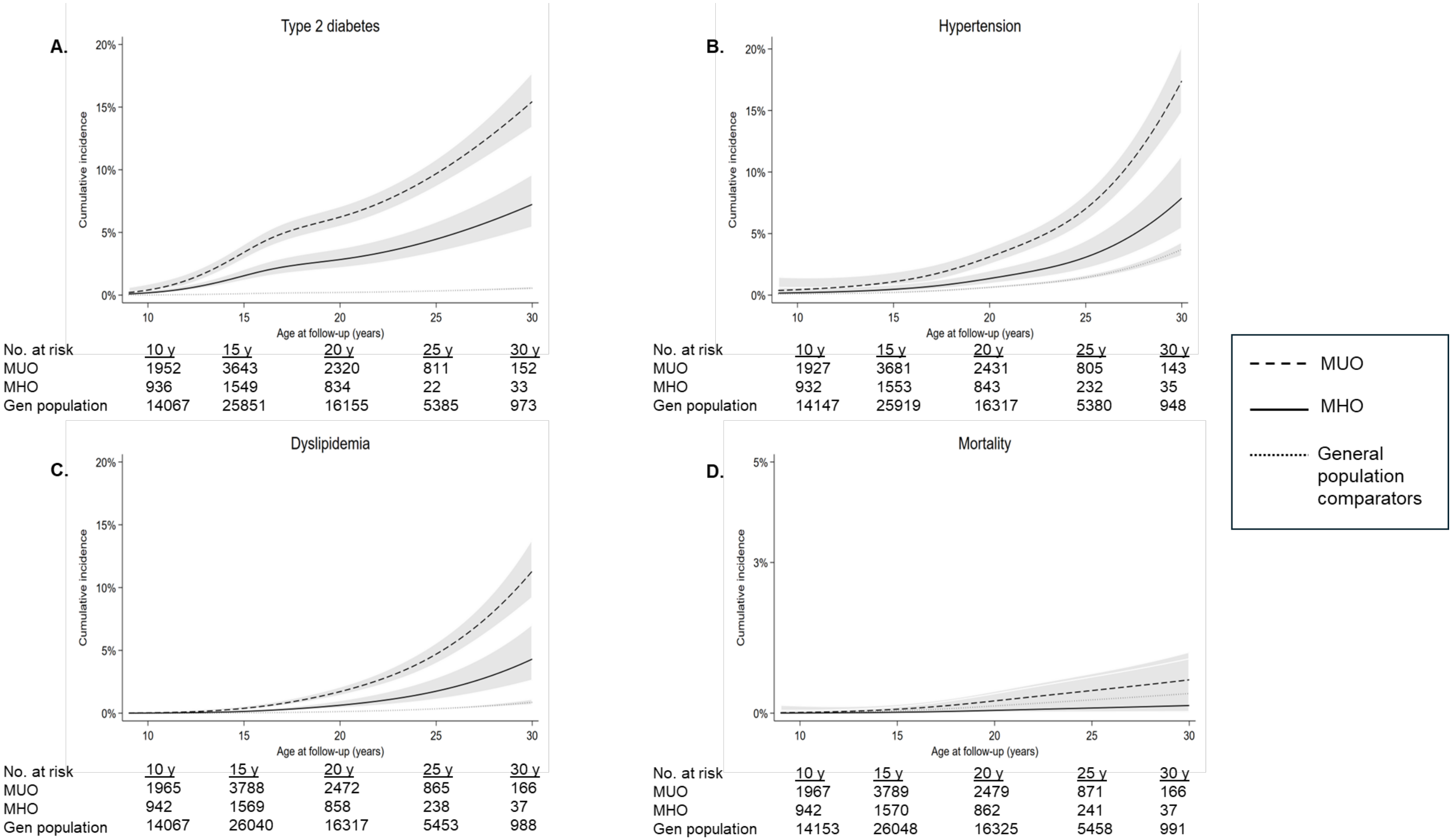

© 2026 Putri RR et al. *JAMA Pediatrics*.
