# Supplementary material for: Long-Term Cardiometabolic Outcomes in Children With Metabolically Healthy and Unhealthy Obesity
Source: JAMA Pediatr. 2026 Mar 23;180(7):750–7. doi: 10.1001/jamapediatrics.2026.0343 (PMC13010205; doi:10.1001/jamapediatrics.2026.0343)
Supplement: Supplement 2. — Data Sharing Statement. [file jamapediatr-e260343-s002.pdf]

Data Sharing Statement

Putri. Long-Term Cardiometabolic Outcomes in Children With Metabolically Healthy and Unhealthy Obesity. *JAMA Pediatr*. Published March 23, 2026.
doi:10.1001/jamapediatrics.2026.0343

Data
Data available: No

Additional Information
Explanation for why data not available: The data used in this study are de-identified individual-level data from Swedish healthcare registers and can be obtained from the respective Swedish data holders, subject to ethical approval for the research question, relevant legislation, and data protection processes.
